# Geographical distribution of mobile genetic elements in microbial communities along the Yucatan coast

**DOI:** 10.1371/journal.pone.0301642

**Published:** 2024-04-29

**Authors:** Francisco Guillén-Chable, Johnny Omar Valdez Iuit, Luis Alejandro Avila Castro, Carlos Rosas, Enrique Merino, Zuemy Rodríguez-Escamilla, Mario Alberto Martínez-Núñez

**Affiliations:** 1 UMDI-Sisal, Facultad de Ciencias, Universidad Nacional Autónoma de México, Sisal, Yucatán, México; 2 Departamento de Microbiología Molecular, Instituto de Biotecnología, Universidad Nacional Autónoma de México, Cuernavaca, Morelos, México; 3 Facultad de Sistemas Biológicos e Innovación Tecnológica, Universidad Autónoma "Benito Juárez" de Oaxaca, Oaxaca de Juárez, Oaxaca, México; 4 UAET-Oaxaca, Instituto de Geografía, Universidad Nacional Autónoma de México, Oaxaca de Juárez, Oaxaca, México; Mississippi State University, UNITED STATES

## Abstract

Horizontal gene transfer (HGT) is a well-documented strategy used by bacteria to enhance their adaptability to challenging environmental conditions. Through HGT, a group of conserved genetic elements known as mobile genetic elements (MGEs) is disseminated within bacterial communities. MGEs offer numerous advantages to the host, increasing its fitness by acquiring new functions that help bacteria contend with adverse conditions, including exposure to heavy metal and antibiotics. This study explores MGEs within microbial communities along the Yucatan coast using a metatranscriptomics approach. Prior to this research, nothing was known about the coastal Yucatan’s microbial environmental mobilome and HGT processes between these bacterial communities. This study reveals a positive correlation between MGEs and antibiotic resistance genes (ARGs) along the Yucatan coast, with higher MGEs abundance in more contaminated sites. The Proteobacteria and Firmicutes groups exhibited the highest number of MGEs. It’s important to highlight that the most abundant classes of MGEs might not be the ones most strongly linked to ARGs, as observed for the recombination/repair class. This work presents the first geographical distribution of the environmental mobilome in Yucatan Peninsula mangroves.

## Introduction

Bacterial organisms have a great functional diversity that allows them adapt to different environments. This functional diversity derives from the plasticity of its genome, which is enriched not only by the transfer of genetic material from parents to daughter cells, but also by transfer of genetic information between individuals, in an ancient process called Horizontal Gene Transfer (HGT) [1–3]. HGT has been documented as a general strategy that stimulates adaptability to adverse environmental conditions, providing a set of molecular functions that facilitate the survival of bacterial species and their communities [4, 5]. HGT has been associated with the spread of a group of conserved genetic elements known as Mobile Genetic Elements (MGEs) within bacterial communities [6, 7]. MGEs are structurally and mechanistically distinct types of elements, and have been cataloged into five categories: the first class, integration/excision (I/E), includes elements that can integrate into the host genome and then excise themselves. The second class, phages, are viruses that can infect bacteria. The third class, recombination/repair (R/R), includes elements that can facilitate recombination or repair of the host genome. The fourth class, stability/defense (S/D), includes elements that can help the host genome to maintain its stability or defend itself against infection. The fifth class, transfer, includes elements that can facilitate the transfer of genetic material between bacteria [8]. Each category represents a family of MGEs, and their composition and distribution in organisms is quite diverse and highly variable between species [9]. The mobility of these elements comes from a subset of enzymes such as recombinases, nucleases and transposases, which catalyze the mobilization of stretches of DNA, including double-strand breaks, phosphate bond breaks, and repair strategies [10, 11]. These enzymes, in addition to being highly conserved in bacterial species, can also be transferred to higher organisms [12, 13]. Several studies have documented the fact that MGEs offer a variety of advantages to the host, such as increasing fitness by conferring new or additional functions. For instance, MGEs have the capacity to carry genes that confer resistance to various adverse conditions, including exposure to heavy metals, antibiotics, and plastic contamination. Additionally, they conferring tolerance under diverse environmental conditions, such as varying salinity levels and temperatures [14–16]. Although the MGEs can disperse beneficial characteristics, in some cases the acquisition of the MGEs can be harmful or even fatal for some bacteria or species. In fact, some MGEs can be considered selfish mobile elements that float from one genome to another just to spread their sequence [17, 18]. The distribution of MGEs depends on the bacterial community and the interaction of the species that compose it, as well as the energy cost of maintaining and mobilizing these elements. Furthermore, environmental conditions and interaction with other eukaryotic organisms can modulate the spread of MGEs [19]. The entire set of mobile genetic elements in a genome is known as a mobilome. Mangroves and coasts represent highly dynamic ecosystems, and the organisms present in these places are constantly exchanging information, molecules and signals to face and adapt to a wide variety of environmental and pollution factors [20, 21]. Among the adaptive traits that MGEs gives to the bacterial communities present in the mangroves are genes encoding antibiotic and heavy metals resistance, as well as xenobiotic degradation genes [5, 22, 23], and this is stimulate by the selective pressure exercised by contamination.

Yucatan is a Mexican state situated in the southeast part of the Gulf of Mexico, on the Yucatan Peninsula. This region is characterized by a limestone platform covering approximately 39,524.4 km^2^, and includes 365 km of coastline along the Gulf of Mexico with a strip that reaches up to 20 km inland from the coastline [24]. The Yucatan coast has 54.4% of the mangroves in Mexico and it lacks bodies of surface-water, instead presenting a system of groundwater that flow into the coast [25]. During the last decade, the Yucatan coast has suffered an increase in pollution due to agriculture and livestock activities, industrial discharges, excessive fishing and tourism, negatively impacting ecological, socioeconomic, and health levels [26, 27]. This ecosystem acts as a terminal basin for emerging pollutants generated inland, where in addition to conventional pollutants such as pesticides, polyaromatic hydrocarbons (PAHs) and heavy metal, antibiotic resistance genes (ARGs) are becoming more prevalent [28]. To the best of our knowledge, there has been no prior investigation into the microbial environmental mobilome within the Yucatan coastal zone. Consequently, a complete absence of information exists regarding the processes of horizontal transfer among the bacterial communities inhabiting this locale. Additionally, the geographic dispersion and numerical prevalence of these MGEs remain entirely uncharted. Hence, it is necessary to undertake an assessment of the mobilome’s environmental presence in the coastal zone of Yucatan, in order to have a better understanding of the processes that originate adaptive traits in microbial communities and their possible impact on human and environmental health. The objective of this work was to identify the presence of mobile genetic elements, their abundance and differences in the microbial communities that inhabit the Yucatan coast. To the best of our knowledge, this is the first report to provide a comprehensive analysis on the mobilomes of microbial communities that inhabit the coastal zone of Mexico, in conserved and impacted areas, for which a metatranscriptomic analysis was used. Through this approach we have generated a more detailed picture of the geographic distribution of the environmental microbial mobilome that is present on the Yucatan coast.

## Material and methods

### Site description and sample processing

The comparison of the microbial communities present in the sediments of four wetlands with different degrees of anthropogenic impact in the Yucatan Peninsula was made by selecting the contaminated sites of the Sisal (21°09’43.6” N; 90°02’27.2” W) and Progreso (21°16′37.6′′N; 89°40′35.6′′W) swamps. And the conserved sites of the ecological reserve of El Palmar (21°08’56.4” N; 90°06’07.0” W) and Bocas de Dzilam (21°27′22.2′′N; 88°40′53.7′′W). Three sediment samples were taken for each site, for which three points were chosen within a box meter square: one point at the center and two more at the ends. The samples were extracted approximately 20 cm deep, and 2 g of sediment were taken from each point and mixed with 6 mL of the LifeGuard Soil Preservation buffer (Qiagen, Hilde, Germany) in 50 ml sterile polypropylene tubes and stored at −20°C. The experiment was conducted at three different times, one in May 2017, March 2018 and October 2019 (S1 Table). No ethical permission was required to carry out the study.

### Nucleic acids extraction and transcriptomic sequencing

Nucleic acids extraction, libraries construction and sequencing were requested from the Research and Testing Laboratory (Lubbock, TX, USA). For RNA extraction Qiagen RNeasy PowerSoil Total RNA Kit (Qiagen, Hilde, Germany) was used following the manufacturer’s recommendations. RNA sequencing libraries were constructed and sequenced following a default Illumina Stranded Total RNA Prep protocol. Sequencing was done using an Illumina HiSeq 2500 platform to generate 2 × 150 bp paired-end reads. Sequencing resulted in an average yield of 58 million reads per sample.

### Metatranscriptomics data analysis

The raw data obtained from the sequencing by RNA-seq of the triplicates for each site were first filtered to remove adapters, as well as low-quality reads (Phred score<30), using the NGS QC Toolkit v2.3.3 software [29], and its program IlluQC.pl for Illumina data using default parameters. A library of Palmar samples was removed due to the low number of reads obtained, staying at the end with 11 libraries: 2 libraries for El Palmar site, and 3 libraries for the rest of the 3 sites. Subsequently, the filtered reads were assembled by *de novo* assembly package Trinity [30]. Annotation of assembled sequences was performed locally using BLASTX [31, 32] sequence similarity searches against the protein UniProtKB/Swiss-Prot database [33], with a threshold of e-value < 10^−20^.

### Mobile genetic elements analysis and annotation

Functional annotation of MGEs present in our metatranscriptomics data was conducted using the MGEfams and mobileOG-db databases. MGEfams database comprises hidden Markov models (HMMs) extracted from Pfam (v 30.0) and TIGRFAM, based on matching strings in their functional annotations using specific keywords [34]. These keywords include terms such as "transposase," "transposon," "conjugative," "integrase," "integron," "recombinase," "resolvase," "conjugal," "mobilization," "recombination," and "plasmid". The HMMs identified as MGEs are categorized into six classes by MGEfams: integrase, revolvase, plasmid, recombinase, conjugation and transposase. We conducted a search for MGEs within our metatranscriptomics data using the hmmscan program from the HMMER v3.3.2 software [35], employing the profiles from the MGEfams v0.5 database. Sequences identified as MGEs were those that had a bit score > 50 from any domain belonging with one of the six classes of MGEs annotated in MGEfams. Additionally, a second search strategy for MGEs was carried out utilizing the hand-curated mobileOG-db Beatrix 1.6 v1 database. This database encompasses protein families that mediate the life cycle of bacterial mobile genetic elements, categorizing them into five classes: integration/excision, recombination/repair, phages, transfer and stability/defense [36]. The identification of MGEs entailed conducting sequence alignments between our transcripts and the mobileOG-db database using the BLASTp program [31]. The search was performed locally, using a threshold e-value < 1e^−20^ to select those sequences identified as MGEs. For the final assignment of MGE classes to our transcripts, we decided to use the classification provided by the mobileOG-db database. When discrepancies arose in the MGE class assigned to the analyzed transcripts between the MGEfams and mobileOG-db databases, we gave priority to the classification provided by mobileOG-db. Likewise, in cases where both databases assigned the same classes, we adhered to the mobileOG-db classification. Therefore, the transcripts identified as MGEs were categorized into one of the five classes of MGEs provided by mobileOG-db, as other studies have done [37, 38]. The RSEM v.1.3.3 package [39] was used to normalize the reads data to correct for library size and gene lengths bias, in order to compare count of genes among the samples from the study sites. Through RSEM we obtained the transcripts per million (TPM) values and these were used to extract MGEs counts that were unique to each site. A custom Perl script was used to extract the unique MGEs, considering as expressed only those genes with an average TPM ≥ 1.

### Prediction of the association of MGEs to resistance to antibiotics

The Comprehensive Antibiotic Resistance Database (CARD) v.3.2.0, which providing reference DNA and protein sequences of bacterial antimicrobial resistance and also mobile genetic elements [40], was used to classify and quantify the antibiotic resistance genes associated with the sequences annotated as MGEs resulting from the previous analysis. A local BLASTx [31, 32] of the MGEs sequences was performed against the CARD database to identify antimicrobial resistance associated with MGEs. The sequences of mobile elements that had a hit in CARD database with a similarity over 90%, an alignment longer than 25 amino acids and e-value of 1e^-20^, were associated with an antibiotic resistance classes. The MGEs associated with ARGs were classified into 60 classes of antibiotic resistance, and 7 mechanisms of resistance.

### Operon reconstruction

To carry out the reconstruction of the operons associated with MGEs, we used the Gene Context Tool NG program [41]. We entered the Swiss-Prot ID associated with each identified MGE into the Gene Context Tool to identify whether the mobile element was associated with any operon. We manually selected three of the most abundant transcripts predicted to be MGE per site location and examined whether they were located within an operon context.

### Visualization and statistical analysis

To carry out the visualization and the statistical analysis of the data, the free software environment for statistical computing and graphics R v.4.2.1 was used [42]. The ggplot2 package (version 3.3.6) was used to build the tables and figures presented here. Circular layout representation of our data regarding the MGE classes and taxonomic clades were constructed with *circlize* package (version 0.4.15), using the dependency Chord-Diagram [43]. The statistical significance of the difference in the number of MGEs between the samples was performed using the Kruskal-Wallis Rank Sum and Pairwise Wilcoxon Rank Sum Tests using the *stats* v.4.3.0 package of R. Correlation between MGEs and ARGs was evaluated with *stat_cor* function using Pearson coefficient. The map was created with the QGIS 3.26 "Buenos Aires" software and the public domain map database Natural Earth (http://www.naturalearthdata.com/).

## Results

### Distribution and composition of mobile genetic elements along the Yucatan coast

**Fig 1:**
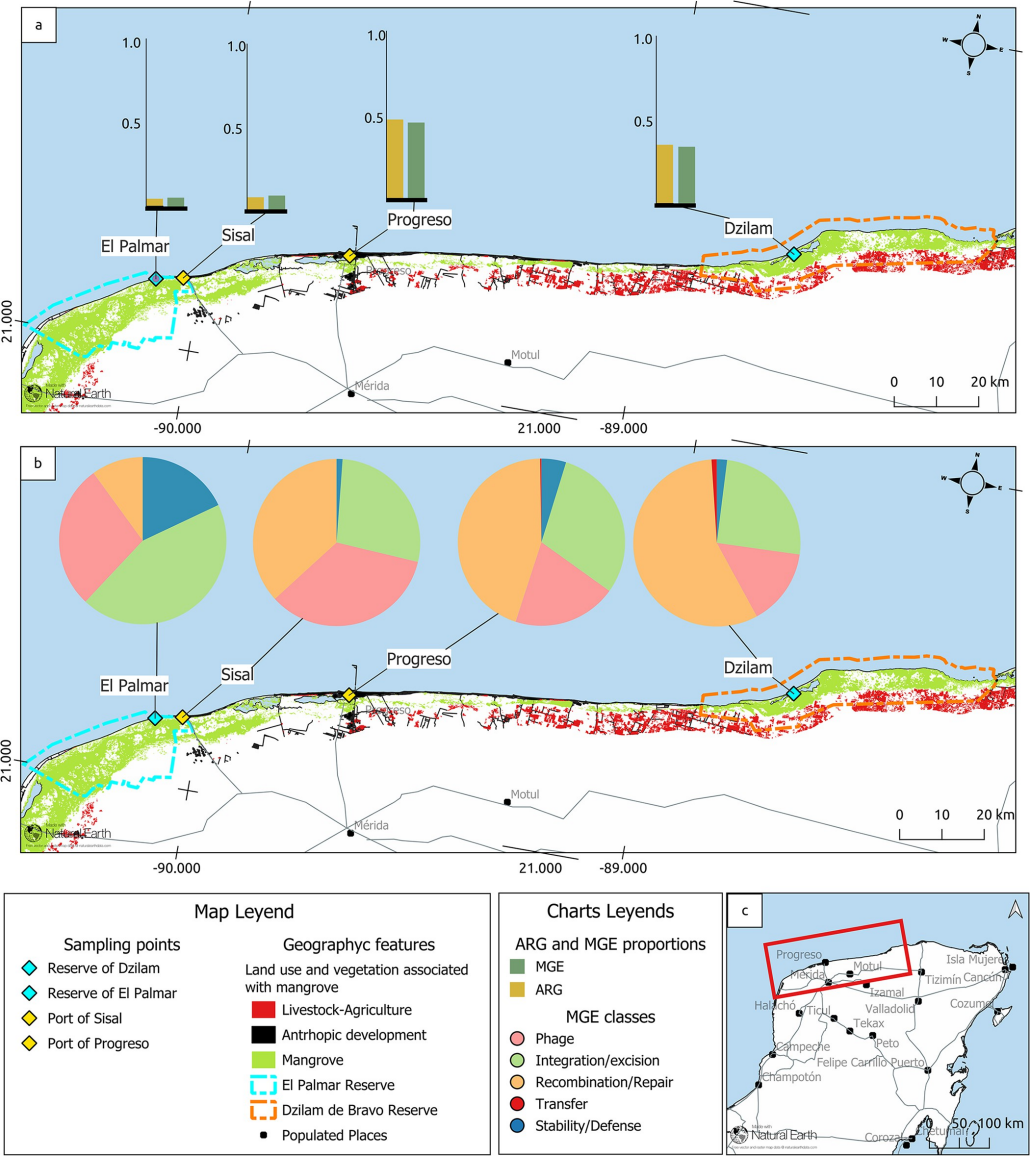
Abundance and geographical distribution of MGEs classes per sampled site. (a) Abundance of total transcripts identified as MGEs and total transcripts identified as ARGs (Guillen-Chable et al., 2022) in sample sites. (b) Distribution of the five classes of MGEs in the samples sites of Palmar, Sisal, Progreso y Dzilam. (c) Study area, Yucatan coast.

To determine the distribution, composition, and abundance of MGEs in microbial communities in the Yucatan Peninsula, we conducted a massive sequencing analysis of their transcriptomes. Samples were taken from four sites along the Yucatan coast: two conservation sites and two affected by anthropogenic activities (Fig 1C). After assembly of the transcriptome reads, we used the mobile element databases MGEfams v0.5 [34] and mobileOG-db Beatrix 1.6 v1 [36] to identify the MGEs present in the transcripts of bacteria inhabiting the Yucatan coast (Fig 1A). This resulted in the identification of five classes of mobile elements according to the mobileOG-db classification: integration/excision (I/E), phages, recombination/repair (R/R), stability/defense (S/D), and transfer (Fig 1B; S2–S5 Tables). The distribution of the total transcripts identified as MGEs in the conserved sites of Palmar and Dzilam was 50 and 202, respectively. For Sisal and Progreso, both human impacted sites, we found 87 and 398 total transcripts identified as MGEs, respectively (Fig 1A). The composition of the MGEs was not homogeneous in the analyzed sites. In the case of the Progreso and Dzilam sites, 5 classes of MGEs were identified; while in the Palmar and Sisal sites, only 4 classes were identified (Fig 1B). The abundance of the classes of MGEs was not homogeneous either. The transfer class was the least represented of all, with a relative abundance of 0.25% and 0.99% only in Progreso and Dzilam, respectively. The recombination/repair class was the most abundant in 3 of the 4 sampled sites, with the maximum value in Dzilam (56.93%), followed by Progreso (44.72%) and Sisal (36.78%). At the Palmar site, the recombination/repair was the least abundant class with 10% (Fig 1B). The phage and integration/excision classes were present at all sites. It should be noted that within the phage class, plasmid sequences can also be found. As outlined in the methodology, we adopted the mobileOG-db classification. In cases where a transcript was labeled as a plasmid by MGEfams and as a phage by mobileOG-db, we prioritized the latter classification due to its stricter criteria. This bias in transcript classification may result in an underrepresentation of plasmid abundance in the microbial communities of the Yucatan coast, as well as their ecological role in HGT processes. However, the distinction between both types is blurred by the existence of elements known as phage-plasmids, which possess attributes of both plasmids and phages [44, 45]. Therefore, developing more accurate classification methods for plasmid and phage elements is imperative. The relative abundance of phage and integration/excision classes decreased in a general geographic pattern running from west to east. The phage class was most abundant in Sisal (34.48%), followed by Palmar (28%), Progreso (20.1%), and Dzilam (14.85%). The integration/excision class showed a similar pattern, with the highest abundance in Palmar (44%), Progreso (30.15%), Sisal (27.58%) and the lowest abundance in Dzilam (25.24%) (Fig 1B). Finally, stability/defense class was present in all sites with the maximun value in Palmar (18%), followed by Progreso (4.77%), Dzilam (1.98%), and lastly Sisal (1.14%). Broadly speaking, the prevalence of MGEs within the bacterial communities situated along the Yucatan coast follows a discernible pattern of increment from west to east (Fig 1A).

### Positive correlation between MGEs and ARGs in the Yucatan Peninsula

**Fig 2:**
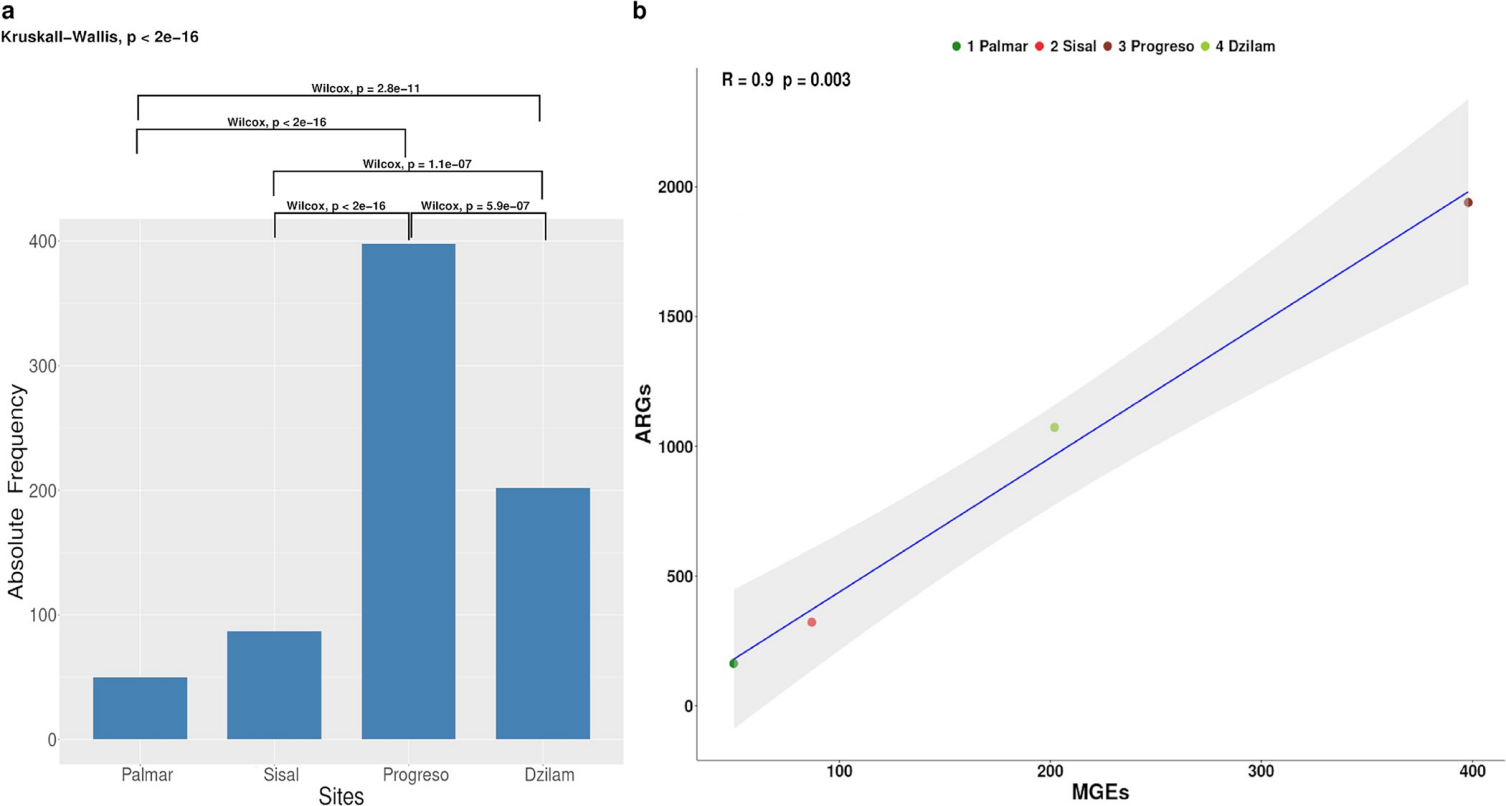
Abundance of total MGEs and correlation with ARGs per sampled site. (a) Kruskall–Wallis and Wilcox test of MGEs abundance in the sampled sites. (b) **Correlation Pearson test** for assessing the relationship between abundance of MGEs and ARGs across study sites.

The total number of MGEs identified on the Yucatan coast showed a geographic pattern, with an increase in abundance from west to east. This pattern has also been observed for antibiotic resistance genes (ARGs) present in microbial communities of the Yucatan coast (Fig 1A) [28]. In order to evaluate the existence of differences in the abundance of MGEs at each site, a Kruskall-Wallis test was performed. The statistical result showed a difference (*p*-value < 2.0*e*–16) between the abundance levels of the samples. To identify which groups presented a significant difference in the content of MGEs, we performed a paired Wilcox test. We found a significant difference (p-value < 0.05) between all the groups, except for the samples of Palmar and Sisal (Fig 2A). To determine the possible existence of a positive correlation between the abundance of MGEs and ARGs in the analyzed sites, we performed a Pearson correlation test. The result revealed a positive linear correlation between the two variables. This signifies that both elements exhibit a coordinated increase, following a geographical trend from west to east (Fig 2B).

### Exploring the genomic organization of MGEs within bacterial communities

**Fig 3:**
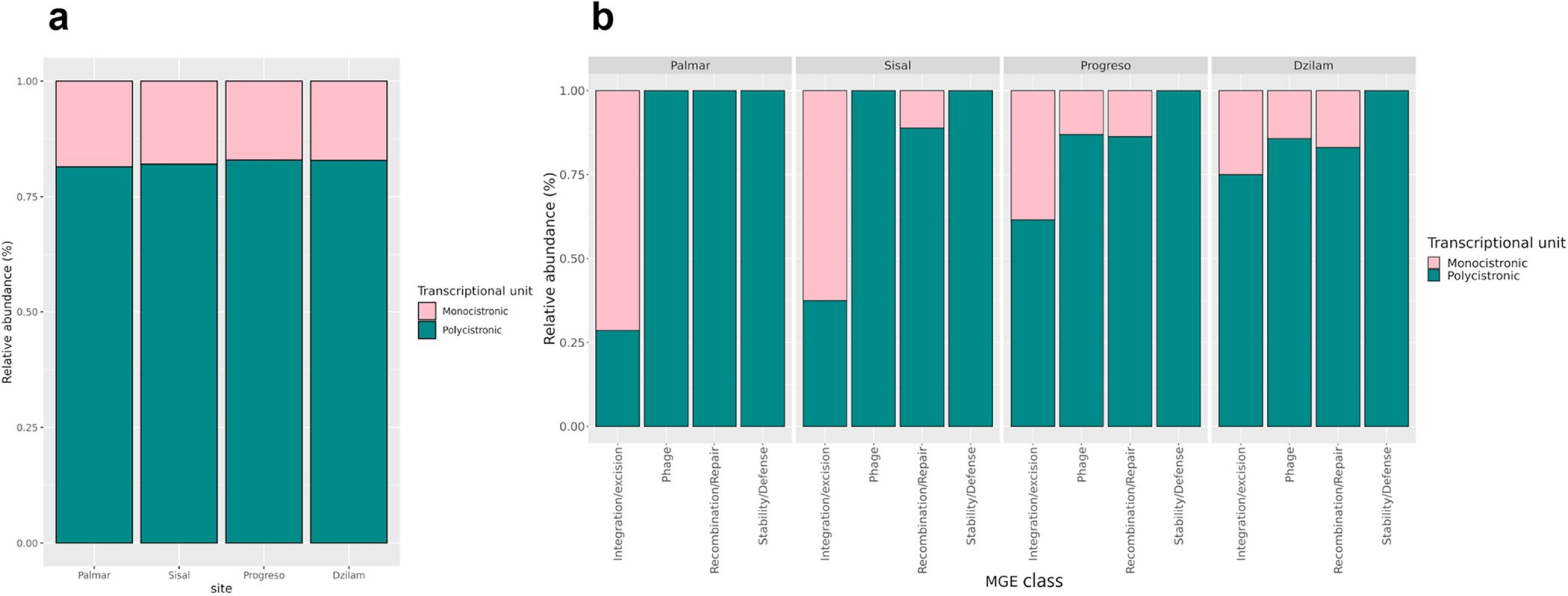
Distribution of MGE classes by integration in the bacterial genome as monocistronic or polycistronic units. (a) Distribution of total MGEs in monocistronic and polycistronic units per site. (b) Distribution of MGE classes in monocistronic and polycistronic units per site.

To gain insights into how MGEs are integrated into the bacterial genomes of the Yucatan coast, we explored their transcriptional organization. We classified them into monocistronic or polycistronic units based on their genomic context. This classification depended on whether the MGEs were transcribed individually or in coordination with other genes. Fig 3A provides a summary of our findings regarding how total MGEs are integrated into bacterial genomes across the sampled sites. It reveals a consistent pattern of MGEs insertion into polycistronic units in all the sampled sites, with 75% of mobile elements being located within operons. The remaining 25% of MGEs are found in monocistronic units. When examining the genomic organization of MGEs by their classes, the same common trend emerges, which is their presence within operons across all classes (Fig 3B). The integration/excision class is the only exception, with a significant portion of its elements organized in a monocistronic arrangement. This monocistronic pattern is particularly notable in sites located to the west of the Yucatan coast, such as Palmar and Sisal. In fact, 60% of elements in this class are expressed in a monocistronic manner (Fig 3B). However, as one moves towards the east, this percentage gradually declines, dropping to less than 40%. On the other hand, the stability/defense class is the sole category of MGEs consistently located within operons. Regardless of the sampling site, this class consistently exhibits polycistronic transcriptional expression. Regarding the phage and recombination/repair classes, their genomic arrangement shows minimal variation, with most elements organized in operons, particularly in sites on the western coast (Fig 3B). However, as we move eastward, these classes exhibit an increasing percentage of their elements in monocistronic arrangements. This classification into monocistronic and polycistronic units highlights a common trend of MGEs incorporating into operons, particularly in the stability/defense class.

### Exploring the MGEs classes in bacterial species from the Yucatan coast

**Fig 4:**
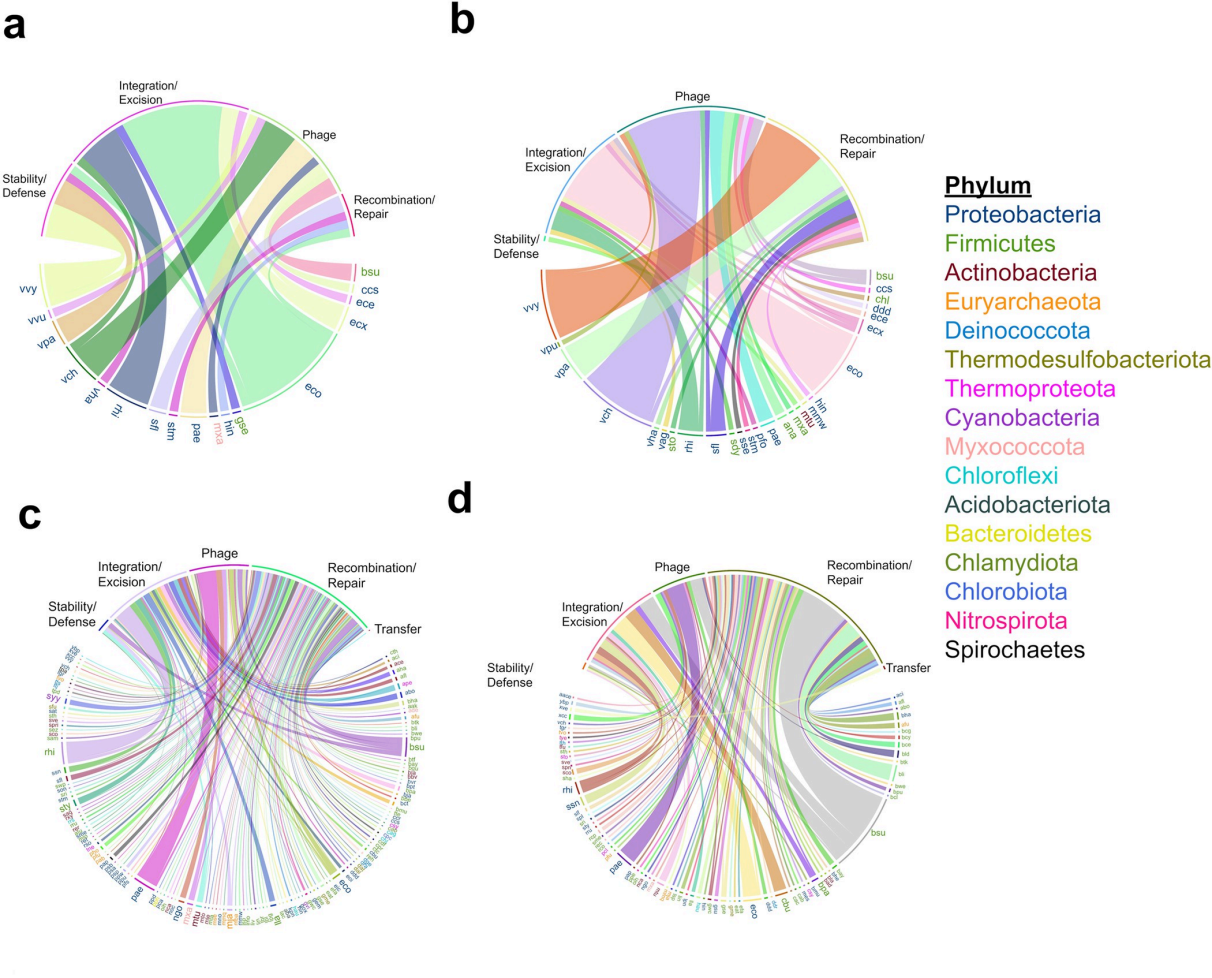
Distribution of MGEs classes per specie and sampling location. MGEs classes associated with the species found at the (a) Palmar, (b) Sisal, (c) Dzilam, and (d) Progreso sites.

To learn more about the presence and abundance of MGEs identified among the bacterial species inhabiting the studied sites, we quantified each class of mobile element in the different bacterial species identified. Based on our dataset, we identified a total of 173 distinct bacterial species spread across the four sampled locations, which exhibited the presence of MGEs. The content of the MGEs varies according to the species and place of origin, which suggests a variation possibly related to human activities developed in the sampling sites. The phyla with the high abundance of MGEs classes were Proteobacteria (44%), Firmicutes (24%) and Actinobacteria (8%). Other less abundant phyla were Chloroflexi (2%), Cyanobacteria (1.7%), Myxococcota (1.1%) and Acidobacteria (0.57%). The aforementioned phyla were heterogeneously distributed in the analyzed sites. In Palmar, only the phyla Proteobacteria, Firmicutes, and Myxococcota were identified, and 17 species were distributed among them. The four classes of MGEs identified at this site were mainly associated with Proteobacteria species. Some examples of these Proteobacteria species include *Escherichia coli* K12 (eco), *Vibrio vulnificus* YJ016 (vvy), *Vibrio cholerae* serotype O1 (vch), and *Sinorhizobium fredii* (rhi). In the case of Firmicutes, the two species belonging this phylum, *Bacillus subtilis* 168 (bsu) and *Geobacillus stearothermophilus* (gse), were associated with phage and integration/excision classes, respectively. On the other hand, *Myxococcus xanthus* DK 1622 (mxa), belonging to the Myxococcota phylum, presented the phage class (Fig 4A). Similarly, in Sisal, only three phyla were identified: Proteobacteria, Actinobacteria, and Firmicutes. Within these phyla, 26 species were distributed among them. The four classes of MGEs at this site were mainly distributed among Proteobacteria species, including *V*. *vulnificus*, *Vibrio parahaemolyticus* serotype O3:K6 (vpa), *V*. *cholerae* serotype O1, *S*. *fredii*, and various strains of *E*. *coli*. The Actinobacteria phylum had only one specie, *Mycobacterium tuberculosis*, which is associated with the stability/defense class (Fig 4B). In Progreso and Dzilam, a greater variety of phyla was identified, and the number of bacterial species distributed within them was greater compared to the Palmar and Sisal sites. The five MGEs classes identified at these sites were mainly associated with species belonging to Proteobacteria and Firmicutes phyla. Examples of bacterial species associated with different classes of MGEs in Progreso include *S*. *fredii*, *E*. *coli*, *B*. *subtilis* and *Pseudomonas aeruginosa* ATCC 15692 (Fig 4C). While for Dzilam, some examples of bacteria associated with a large number of MGEs are *B*. *subtilis*, *E*. *coli*, *Coxiella burnetii* RSA 493 (cbu), *P*. *aeruginosa*, *S*. *fredii* and *Shigella sonnei* (ssn) (Fig 4D). In Palmar, 64.7% of the species harbor mobile elements from the phage and integration/excision classes, whereas the remaining 35.3% of the species are linked to the stability/defense and recombination/repair classes. And in Sisal, the majority of species (80.8%) are associated with the phage and recombination/repair classes, while the remaining 19.2% of species exhibit the stability/defense and integration/excision classes. Conversely, in Progreso and Dzilam, genetic exchanges in most species at these sites are primarily mediated by recombination/repair and integration/excision mechanisms.

### Antibiotic resistance carried by MGEs

**Fig 5:**
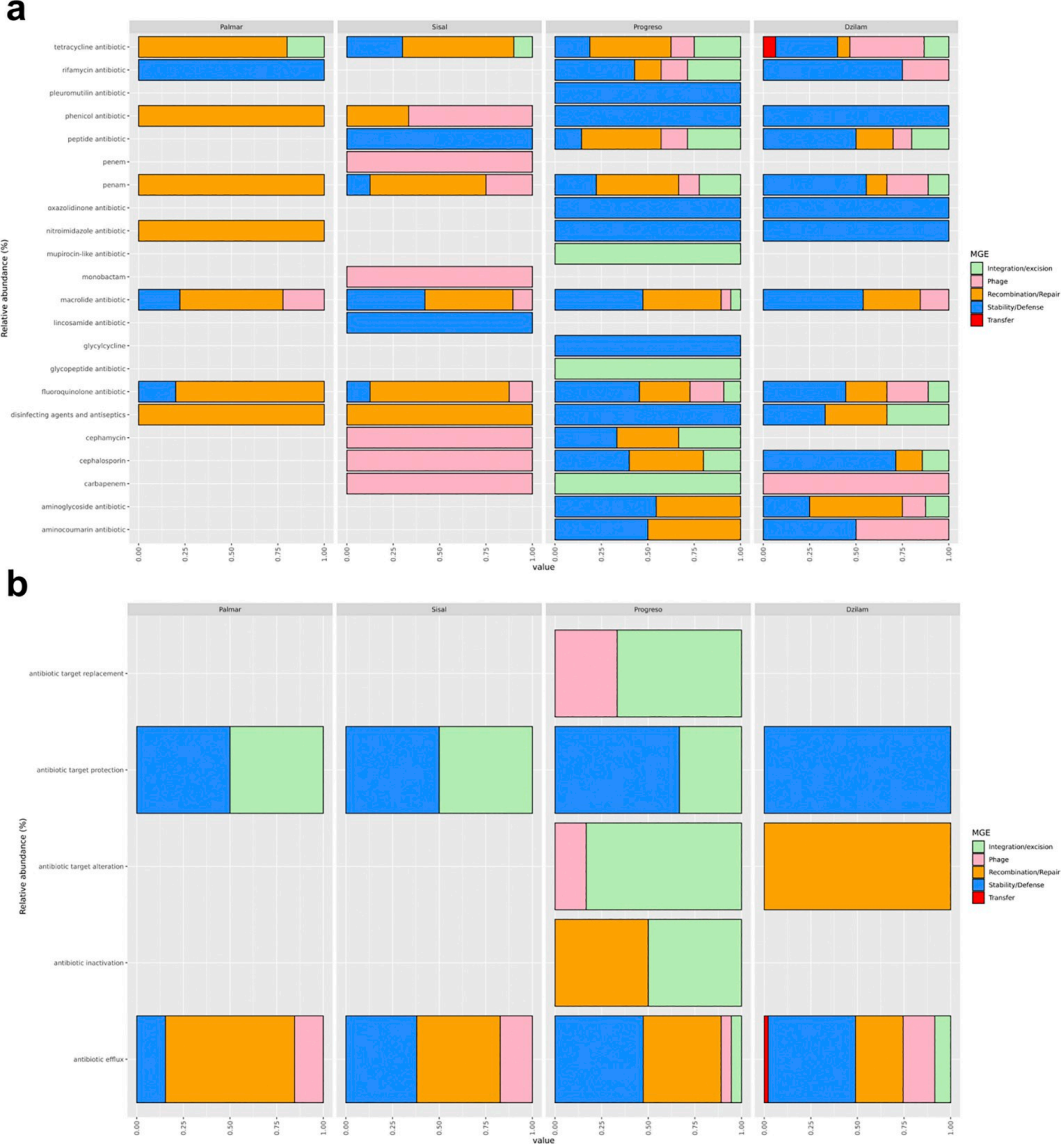
ARG classes and their mechanisms of action associated with MGE. (a) Distribution of ARG classes associated with MGEs by study site. (b) Distribution of antibiotic resistance mechanisms associated with MGEs by study site.

In order to know the role of the MGEs in the dispersion of the ARGs present in the bacterial communities of the Yucatan coast, the identification of the resistance genes classes associated with the mobile elements present in our data was carried out. The identification of the ARGs classes and their resistance mechanisms associated with the sequences previously described as MGEs was carried out using the Comprehensive Antibiotic Resistance Database (CARD v3.2.7) [40]. Out of the 737 transcripts that were initially identified as MGEs, only 190 were found to be linked to 22 distinct ARG classes, along with their respective resistance mechanisms (S6–S9 Tables). The distribution and abundance of MGEs associated with the ARGs in the analyzed sites was as follows: Palmar (7.8%), Sisal (23.7%), Progreso (40.5%), and Dzilam (27.9%). The top ten most abundant classes of ARGs associated with MGEs were: macrolide, tetracycline, fluoroquinolone, penam, peptide, aminoglycoside, cephalosporin, rifamycin, phenicol, and disinfecting agents/antiseptics antibiotics. Fig 5A shows that the ARG classes from the Palmar and Sisal sites primarily exhibit associations with mobile elements related to replication/reparation and phages. Whereas the ARG classes from the Progreso and Dzilam sites are predominantly linked to mobile elements associated with stability and defense. Furthermore, it’s observed that the sites in the eastern region display a higher diversity of ARG classes and MGEs compared to the sites in the western region. For example, at the Progreso and Dzilam sites there are ARG classes such as tetracycline, rifamycin, peptide antibiotic, penam, macrolide antibiotic or fluoroquinolone, which are associated with at least 4 classes of MGEs. Conversely, at the Palmar and Sisal sites, the ARG classes of phenicol, nitroimidazole, carbapenem or cepamycin are associated with a single MGE class. Concerning the mechanisms responsible for antibiotic resistance that are associated with MGEs, our analysis revealed the presence of five distinct types. These mechanisms were distributed as follows among the 190 MGEs: antibiotic efflux (88%), target protection (4%), target alteration (2%), target replacement (2%), and antibiotic inactivation (2%). Among the four sites we examined, only two antibiotic resistance mechanisms were consistently found across all: antibiotic target protection and antibiotic efflux mechanisms (Fig 5B). Antibiotic target protection was exclusively associated with the mobile elements of integration/excision and stability/defense. In contrast, the antibiotic efflux mechanism displayed a wider range of associations with various MGEs across all sites. Regarding the antibiotic target alteration mechanism, it is noteworthy that its occurrence in Dzilam is linked exclusively to the mobile element related to recombination/repair. Similarly, mirroring the pattern observed with ARG classes, the Progreso site stands out as having the highest diversity of MGEs associated with the identified antibiotic resistance mechanisms.

## Discussion

### Geographical pattern of the MGEs and ARGs of the microbial communities of the Yucatan coast

The results of the analysis of bacterial transcriptomes from the Yucatan coast showed that the abundance, composition, and distribution of MGEs were not homogeneous across the four sites. Two observations can be made from the results obtained: firstly, the Progreso sampling site, positioned within one of the five most densely populated cities in Yucatan and thus influenced by significant human activities, exhibits a notably higher abundance of mobile elements when compared to the other sites (Fig 1A). The findings suggests that HGT plays an important role in conferring tolerance to various contaminants to the bacterial communities present in mangroves affected by pollution processes [5, 46]. Furthermore, these observations suggests that polluted mangroves are areas of high transfer of genetic information between bacterial species through MGEs. Consequently, these spaces emerge as scenarios where bacterial communities can adapt to adverse environmental fluctuations [22]. The composition and abundance of each class of MGEs exhibited variations among the different sites. Specifically, in the Progreso and Dzilam sites, we identified a total of five distinct classes of MGEs. In contrast, at the Palmar and Sisal sites, we found only four classes (Fig 1B). This contrasts with the findings of a previous study conducted by Cotta *et al*. (2019) [22], focused on Brazilian mangroves. Their research reported a lower diversity of MGEs, specifically highlighting the presence of phages, plasmids, and transposable elements. In their study, reads representative of phages obtained through metagenomics and metatranscriptomics, were the most prevalent compared to the other two mobile elements. In contrast, our study unveiled a higher diversity of MGEs, encompassing a total of five distinct classes of these mobile elements. Notably, the recombination/repair class stands out as the most prevalent, exhibiting an increase in abundance as we move towards the eastern portion of the coast. Followed in abundance by the phage and integration/excision classes, both with a general decrease towards the east (Fig 1B). Secondly, the abundance of all MGEs increased in a general geographic pattern running from west to east. If we group the sampling sites by their geographical location, the sites that are located to the west of the Yucatan coast, Palmar and Sisal, present a lower proportion of MGEs in comparison with the sampling sites to the east of the coast, Progreso and Dzilam, which present a higher abundance of MGEs (Fig 1A).

The geographic distribution pattern of MGEs along the Yucatan coast aligns with the trend observed for ARGs within the same region. This similarity entails an increase in abundance moving from the western to the eastern regions of the Yucatan coast, as reported by Guillen-Chable *et al*. (2022) [28]. This geographic pattern suggests that there is a strong positive correlation between the abundance of MGEs and the abundance of antibiotic resistance genes in the analyzed sites, as indicated by the Pearson correlation test shown in Fig 2B. This finding is consistent with the hypothesis that the presence of MGEs potentially plays a role in the emergence and dissemination of antibiotic resistance within microbial communities [16, 22, 47] as observed along the Yucatán coast. Jiang *et al*. (2021) [43] previously reported the correlation between MGEs and ARGs in mangrove communities on Hainan Island, China. They used high-throughput quantitative polymerase chain reaction (HT-qPCR) to study the relationship between MGEs and ARGs in these ecosystems. Similarly, Sun *et al*. (2022) [47] conducted a study on mangrove sediments around the provinces of Zhanjiang and Guangdong in China and also reported a high correlation between MGEs and ARGs. They found that this correlation varied depending on environmental and sociometric factors. Our data suggests the existence of a potential gradient of antibiotic resistance along the Yucatan coast. This geographic pattern is likely linked, in part, to varying levels of human activity at the four sites we examined, as well as the presence of submarine groundwater discharge (SGD) in the Yucatan Peninsula. Sites located to the west of the coast are characterized by a relative absence of anthropogenic disturbances, as exemplified by Palmar. In the case of Sisal, this site also exhibit lower population density and reduced human activities when compared to the town of Progreso. Conversely, elevated human activity stemming from higher population densities and intensified livestock activities on the eastern coast may result in the introduction of novel MGEs into microbial communities, as well as an increase in their abundance. In addition, the presence of these karst aquifers in the Yucatan Peninsula has been well-documented for their high capacity to store and transport contaminants from sources to areas of potential exposure, such as are the estuaries. Within these karst aquifers, there is a SGD that crosses the livestock production area in the east (Fig 1A), and could be dragging the antibiotic residues that would reach the Dzilam coast [25, 28], affecting the ecological reserve. These studies provide further evidence of the association between MGEs and ARGs, emphasizing the importance of understanding their dynamics in microbial communities and their potential implications for the development and spread of antibiotic resistance in coastal ecosystems.

### MGEs in coastal bacterial species: Variation in content and distribution

In this study, our objective was to gain insights into the presence of MGEs within bacterial species situated along the Yucatan coast. Our findings unveiled the existence of 173 bacterial species harboring MGEs across the examined sites. Notably, the identified bacterial species are primarily affiliated with phyla that are closely linked to mangrove sediments, including Proteobacteria, Acidobacteria, Chloroflexi, and Firmicutes [48–52]. The content of MGEs in the organisms displayed variability across different phyla and sampling locations within the study sites. Particularly noteworthy is that the phyla Proteobacteria, Firmicutes, and Actinobacteria collectively accounted for the highest abundance of MGEs, representing 76% of all identified MGE classes in this study. This observation agrees with a prior study conducted by Botelho (2023) [53], which also identified Proteobacteria, Firmicutes, Actinobacteriota, Bacteroidota, and Campylobacterota as the phyla with the greatest proportion of MGEs. This highlights the importance of these phyla in both harboring and potentially facilitating the dissemination of MGEs within the analyzed microbial communities. Additionally, less abundant phyla such as Chloroflexi, Cyanobacteria, Myxococcota, and Acidobacteria were also detected, albeit in smaller proportions. In general, phyla that have MGEs exhibit heterogeneous distribution patterns across the four sampling sites. Palmar and Sisal display a less diverse representation of phyla compared to Progreso and Dzilam. Remarkably, in both Palmar and Sisal, the phylum Proteobacteria predominates among species containing MGEs. This observation underscores the substantial role that Proteobacteria play in harboring MGEs within these specific locations. Notably, the higher prevalence of the Proteobacteria phylum in Palmar and Sisal has been previously documented by Navarrete-Euan *et al*. (2021) [54]. In Palmar and Sisal, a notable proportion of the identified species containing MGEs were pathogenic bacteria. These included *Haemophilus influenza*, *Vibrio vulnificus*, *V*. *cholerae* O1, *V*. *parahaemolyticus* serotype O3:K6, *Pseudomonas aeruginosa* PAO1, *Salmonella typhimurium*, *Shigella flexneri*, and *Mycobacterium tuberculosis* [55]. These bacteria are known to cause various diseases, and importantly, some of them, such as *S*. *flexneri* and *M*. *tuberculosis*, exhibit resistance to antibiotics [56–59]. At both Palmar and Sisal sites, phage-related functions are among the most abundant classes of MGEs. This suggests a prevalence of phage-mediated genetic exchange processes within the microbial communities situated on the western side of the Yucatan coast, particularly within pathogen genomes. Most bacterial pathogens contain prophages or phage remnants integrated into bacterial DNA, and in many cases, these represent a large fraction of the strain-specific DNA sequences. These elements contribute to the diversification of the bacterial genome architecture; for instance, they can act as anchor points for genome inversions. As a consequence, phages have emerged as the main suspects in the adaptation of pathogens to new hosts and in the emergence of new pathogens or transfer of ARGs [60–62], as well as in facilitating adaptation to various environmental niches. Unlike Palmar and Sisal, where phage-related functions were highly abundant, the number of species associated with the phage class in Progreso and Dzilam was lower. Instead, genetic exchanges in most species at these sites are primarily mediated by integration/excision and recombination/repair mechanisms. Progreso and Dzilam displayed a greater diversity of phyla and a higher count of bacterial species containing MGEs. This observation implies that these sites host more diverse microbial communities with a wider array of species capable of acquiring and harboring MGEs, possibly owing to a heightened presence of contaminants in the environment on the eastern part of the Yucatan coast. These findings are consistent with the idea that the abundance and distribution of bacterial phyla and species, along with their MGEs, can fluctuate across different geographical locations. This phenomenom was already demonstrated for the bacterial landscape of the Yucatan coast [54], as well as for the ARGs present in this same area [28]. The observed variation highlights the diverse dynamics and genetic exchange processes taking place within the microbial communities of these sites, facilitating the transfer of genetic information among individuals and populations. Such variation can be attributed to several factors, including differing environmental conditions [52, 63], levels of human activity, and the potential introduction of new species from external sources. The presence of MGEs within specific species can also influence the behavior and lifestyle of certain bacteria [64]. Allowing them to adapt to various ecological niches by acquiring new metabolic capabilities, including biosynthesizing capsular polysaccharides, utilizing polysaccharides (*susC* and *susD* genes), and engaging in sporulation (*spo0A* and *spoVG* genes) [65]. Additionally, MGEs may enhance pathogenic, symbiotic, or fitness capacities, beyond conferring resistance solely to antibiotics or heavy metals [66]. Understanding these dynamics is crucial for comprehending the role of MGEs in microbial evolution, adaptation, and potential dissemination of genetic traits, including antibiotic resistance genes.

### Transcriptional organization of MGEs in bacterial genomes along the Yucatan coast

Until now, investigations into the transcriptional organization of MGEs have primarily been conducted at the species level [67–69], with limited understanding at the environmental scale. Moreover, coastal regions lack comprehensive insights into the genomic organization of MGEs. This study provides valuable information in this regard, shedding light on genomic organization of MGEs in coastal bacterial communities. It enhances our understanding of how these elements integrate and function in the environmental context, particularly along the Yucatan coast. Our classification of MGEs into monocistronic and polycistronic units, based on their genomic context, reveals that the majority of MGEs identified in coastal bacteria are integrated into polycistronic units across all sampled sites. The 25% of mobile genetic elements are found in monocistronic units, while the remaining 75% are found within operons (Fig 4A), implying a coordinated mechanism of operation for these elements. The prevalence in polycistronic units enables MGEs to work in concert with other genes, allowing bacteria to flexibly adapt to swiftly changing environmental conditions, including responses to antibiotics and metals in contaminated sites. An illustrative case can be seen in the *Pantoea eucrina* OB49 bacterium, where the merRTPCADE operon in association with the arsRBCH operon appear to have been acquired through MGEs from different bacteria. This acquisition assists in dealing with stress caused by mercury and arsenic [37]. However, there is an interesting exception in the integration/excision class, in which a substantial part of its elements are organized in a monocistronic arrangement. This is particularly prevalent in sites located on the western part of the Yucatan coast, such as Palmar and Sisal, where 60% of the elements of this class are transcribed as monocistronic units (Fig 3B). This might suggest the absence of a selective pressure that favors polycistronic transcription for these specific elements. Some integrases, known as suicide integrases, produce pseudogenes that evolve rapidly at high frequency, resulting in the production of functional genetic variants through protogene recombination [70]. In regions with minimal contamination, the selective forces driving the evolution of resistance-related functions, whether against antibiotics or heavy metals, tend to diminish. Consequently, the emergence of novel genes with functions unrelated to resistance becomes more likely, particularly when integration/excision classes are present in monocistronic transcriptional expression units. As observed in sites along the western part of the coast, where both the abundance and the association of the integration/excision class with ARGs are lower, and the prevalence of monocistronic units of integration/excision is higher.

### Association of MGEs and ARGs on the Yucatan coast

The distribution of MGEs associated with ARGs classes showed variation among the studied sites. Our data highlights that ARGs classes with the highest diversity of linked MGEs were primarily concentrated in the eastern sites, particularly Progreso and Dzilam. At these sites, 50% of ARG classes exhibit associations with at least two MGEs. The ARG classes that exhibit a wider range of associations with various MGEs are typically the ones commonly used in clinical settings and livestock production, such as tetracycline, peptide antibiotics, penams, and fluoroquinolones [71–74]. The observed diversification in the association of MGEs and ARGs along the eastern Yucatan coast seems to be an adaptive response to increased pollution in this region. Previous studies have shown a significant correlation between the abundance of ARGs in coastal microbiomes and the pollution gradient [75]. Therefore, the association and diversity of these elements is influenced by environmental pollution levels. Progreso, one of the five largest cities in Yucatan, plays a significant role in this diversification due to its dense population, driven by indiscriminate antibiotic use and improper disposal by the local population. The presence of emerging contaminants, like antibiotics, is known to trigger the development of various resistance mechanisms, as well as the mechanisms for their spread. This impact is not limited to bacteria in clinical settings but also extends to those inhabiting critical ecological areas, such as mangroves [28, 76, 77]. In contrast, in sites situated along the western coast, like as Palmar and Sisal, a predominant association of 60% of ARG classes with a single MGE is observed. The most prevalent of these MGE classes associated with ARGs are recombination/repair and phage elements (Fig 5A). In our study, phage classification may contain plasmid sequences. Although phages and plasmids are generally perceived as highly distinct, it is recognized that certain elements, referred to as phage-plasmids (P-Ps), possess attributes of both plasmids and phages [44]. Research has shown that the gene repertoire of P-Ps overlaps with that of both phages and plasmids. For instance, these elements encode viral particles and packaging machinery similar to those found in phages, as well as replication initiators and partitioning systems akin to those of plasmids [45]. These P-Ps elements encode accessory functions often found in large MGEs, including genes conferring antibiotic resistance [44]. A recent study highlights the presence of 60 P-Ps containing over 180 ARGs, conferring resistance to cephalosporins, carbapenems, aminoglycosides, fluoroquinolones, and colistin [78]. Our findings at the Sisal contaminated site corroborate this observation, as we observed a complete association between the phage class and the cephalosporin and carbapenem resistance classes. The effects of pollution are less pronounced in the west area, which could be a contributing factor to the reduced diversity of MGEs associated with ARGs. Significantly, two specific classes of MGEs, recombination/repair and stability/defense, exhibit notable characteristics. First, the prevalence of the recombination/repair class of MGEs in the eastern region suggests that these elements are abundant in this area (Fig 1B). However, it is intriguing to note that despite their abundance, these MGEs exhibit low association with ARGs in this region. It is possible that these MGEs primarily serve functions unrelated to antibiotic resistance and have adopted new roles (exaptation) that enhance the host’s ability to survive environmental stressors. Throughout microbial evolution, MGEs often undergo exaptation, a process in which MGEs are repurposed for biologically distinct functions due to their fundamental biochemical utility [79]. This mechanism allows MGEs to adapt and contribute to host survival in various ways. On the other hand, the stability/defense class of MGEs, despite being less abundant (Fig 1B), demonstrates a higher frequency of association with ARGs in the same region (Fig 5a). The increased association of stability/defense class with ARGs suggests that these elements could be playing a pivotal role in the dissemination of antibiotic resistance traits in response to the specific challenges posed by the environment in the eastern Yucatan coast. The observed contrast between the abundance and association of these two MGE classes highlights the complexity of microbial responses to environmental stressors, including contaminants and antibiotics. In terms of geographic space, it’s noteworthy that the Progreso site plays a significant role in hosting a high diversity of MGEs associated with antibiotic resistance mechanisms. This observation aligns with the pattern observed for ARG classes, where Progreso displayed a higher abundance of both MGEs and resistance genes [28]. Additionally, the study reveals two key antibiotic resistance mechanisms that consistently appear across all the analyzed sites on the Yucatan coast: antibiotic efflux and antibiotic target protection. This shared presence emphasizes the significance of these mechanisms in the bacterial response to antibiotics and the adaptability of these resistance strategies across diverse ecological niches. The wide range of associations between the antibiotic efflux mechanism and various MGEs across all sites underscores the adaptability of this resistance strategy (Fig 5B). Efflux pumps enable bacteria to actively expel antibiotics from their cells, reducing intracellular drug concentrations and rendering antibiotics less effective [80]. The broad associations with different MGEs indicate that this mechanism can be easily transferred and disseminated among bacterial populations, potentially contributing to the spread of antibiotic resistance genes. This is in line with what was reported by Imchen and Kumavath (2020) [81] on mangrove sediments, in which efflux pumps covered over one-third of the resistome and were significantly enriched in the human impacted mangrove sediments. In contrast to the widespread associations of the efflux mechanism, antibiotic target protection is linked predominantly with two specific MGE classes, integration/excision and stability/defense. This suggests a more specialized role for target protection in safeguarding resistance mechanisms. The mechanism of target protection involves shielding antibiotic target sites to prevent drug binding and subsequent inhibition of essential cellular functions. The specificity in its associations suggests that target protection may have evolved unique relationships with certain MGEs, likely enhancing the dissemination of specific resistance genes. In summary, the findings regarding the high diversity of MGEs associated with ARG classes and resistance mechanisms in Progreso shed light on the complex dynamics of antibiotic presence in the Yucatan coast, and the MGEs to confer tolerance to various contaminants, as has already been reported [8, 82–84].

It is essential to acknowledge the limitations inherent in our study. Our ability to detect indicators of MGEs and ARGs relies heavily on sequences represented in existing databases. However, it’s crucial to recognize that these databases may not comprehensively represent the diversity of environmental communities, particularly in regions where MGEs have not been extensively studied prior to our investigation. This limitation underscores the need for further research and exploration to enhance our understanding of MGE dynamics and their implications for antimicrobial resistance in coastal environmental settings.

## Conclusions

Antibiotics and emerging contaminants are on the rise, increasing in both abundance and misuse, affecting not only human populations but also environmental conservation areas. This study reveals the geographical distribution and abundance of MGEs along the Yucatan coast present in microbial communities. Our findings reveal a positive correlation between MGEs and ARGs across various geographic sites with varying pollution levels and human activity. This correlation highlights the interaction between microbial communities and environmental factors. Suggesting the potential role of MGEs as key drivers in the spread and persistence of ARGs within microbial populations, particularly in environments subject to increased anthropogenic pressures. We also observed a geographic gradient in the abundance of both elements, running from lower to higher abundance from west to east along the coast. This pattern was especially pronounced in sites with a more significant environmental impact. The taxonomic phyla with the highest abundance of MGEs were Proteobacteria and Firmicutes, which is consistent with findings from previous studies. More than three-quarters of the identified MGEs were located within polycistronic transcriptional units, while nearly a quarter were found within monocistronic transcriptional units. Notably, elements of the integration/excision class were predominantly found in monocistronic units, particularly in less contaminated or more conserved sites, suggesting their potential role in generating new molecular functions through the combination of pseudogenes. A particularly notable observation is that the high abundance of a specific class of MGEs at a site doesn’t necessarily imply a strong association with ARGs, as seen with the recombination/repair class. This class showed high abundance at the Progreso and Dzilam sites but had minimal association with ARGs. Conversely, classes of MGEs with low abundance in certain sites exhibited the strongest associations with ARGs. For instance, the stability/defense class, despite having low abundance in Progreso and Dzilam, showed the highest association with ARGs. Similarly, the recombination/repair class, with low abundance in Palmar, displayed the greatest association with ARGs. This report describes mobilomes from the coasts of Yucatan, Mexico, for the first time, and shows how active mangroves in the Yucatan Peninsula are in terms of transfer of genetic information and its impact on microbial communities. This knowledge is instrumental in the development of effective strategies to monitor and combat antibiotic resistance, addressing this growing threat in both clinical and environmental contexts. Additionally, this research significantly contributes to our comprehension and documentation of environmental pollution processes that pose a threat to coastal ecosystems.

## Supporting information

S1 Table Spatial and temporal details of locations sampled in this study. (DOCX)

S2 Table Transcripts identified as MGE at the Palmar site using the MGEfams database v0.5 and mobileOG-db database. (XLSX)

S3 Table Transcripts identified as MGE at the Sisal site using the MGEfams database v0.5 and mobileOG-db database. (XLSX)

S4 Table Transcripts identified as MGE at the Progreso site using the MGEfams database v0.5 and mobileOG-db database. (XLSX)

S5 Table Transcripts identified as MGE at the Dzilam site using the MGEfams database v0.5 and mobileOG-db database. (XLSX)

S6 Table Transcripts identified as MGE and associated to ARGs using the CARD database at the Palmar site. (XLSX)

S7 Table Transcripts identified as MGE and associated to ARGs using the CARD database at the Sisal site. (XLSX)

S8 Table Transcripts identified as MGE and associated to ARGs using the CARD database at the Progreso site. (XLSX)

S9 Table Transcripts identified as MGE and associated to ARGs using the CARD database at the Dzilam site. (XLSX)
