# Supplementary material for: Geographical distribution of mobile genetic elements in microbial communities along the Yucatan coast
Source: PLoS One. 2024 Apr 29;19(4):e0301642. doi: 10.1371/journal.pone.0301642 (PMC11057721; doi:10.1371/journal.pone.0301642)
Supplement: S1 Table — (DOCX) [file pone.0301642.s001.docx]

**S1 Table.** **Spatial and temporal details of locations sampled in this study.**

| **Sampling site** | **Coordinates** | **Sampling replicates in 1 m^2^ surface** | **Date** |
| --- | --- | --- | --- |
| Antropogenic impacted locations | | | |
| Sisal | 21°09’43.6” N  90°02’27.2” W | 3 | May, 2017 |
| Progreso | 21°16’37.6” N  89°40’35.6” W | 3 | October, 2019 |
| Ecological reserved locations | | | |
| Palmar | 21°08’56.4” N  90°06’07.0” W | 3 | March, 2018 |
| Dzilam | 21°27’22.2” N  88°40’53.7” W | 3 | October, 2019 |
